# A catalog of hemizygous variation in 127 22q11 deletion patients

**DOI:** 10.1038/hgv.2015.65

**Published:** 2016-01-14

**Authors:** Matthew S Hestand, Beata A Nowakowska, Elfi Vergaelen, Jeroen Van Houdt, Luc Dehaspe, Joshua A Suhl, Jurgen Del-Favero, Geert Mortier, Elaine Zackai, Ann Swillen, Koenraad Devriendt, Raquel E Gur, Donna M McDonald-McGinn, Stephen T Warren, Beverly S Emanuel, Joris R Vermeesch

**Affiliations:** 1 Department of Human Genetics, KU Leuven, Leuven, Belgium; 2 Institute of Mother and Child, Warsaw, Poland; 3 Genomics Core, UZ Leuven, Leuven, Belgium; 4 Department of Human Genetics, Emory University School of Medicine, Atlanta, GA, USA; 5 VIB Departement Moleculaire Genetica, University of Antwerp, Antwerp, Belgium; 6 Department of Medical Genetics, Antwerp University Hospital, Edegem, Belgium; 7 Human Genetics, The Children's Hospital of Philadelphia, Philadelphia, PA, USA; 8 Perelman School of Medicine, University of Pennsylvania, Philadelphia, PA, USA

## Abstract

The 22q11.2 deletion syndrome is the most common microdeletion disorder, with wide phenotypic variability. To investigate variation within the non-deleted allele we performed targeted resequencing of the 22q11.2 region for 127 patients, identifying multiple deletion sizes, including two deletions with atypical breakpoints. We cataloged ~12,000 hemizygous variant positions, of which 84% were previously annotated. Within the coding regions 95 non-synonymous variants, three stop gains, and two frameshift insertions were identified, some of which we speculate could contribute to atypical phenotypes. We also catalog tolerability of 22q11 gene mutations based on related autosomal recessive disorders in man, embryonic lethality in mice, cross-species conservation and observations that some genes harbor more or less variants than expected. This extensive catalog of hemizygous variants will serve as a blueprint for future experiments to correlate 22q11DS variation with phenotype.

## Introduction

The 22q11.2 deletion syndrome is the most common microdeletion disorder in humans, with a prevalence of ~1 in 4,000 live births.^1,2^ The 22q11 deletion is for the large majority a result of non-allelic homologous recombination between low copy repeats (LCRs). The most common deletion is ~3 Mb occurring between LCRs 22-A and 22-D (~90%)^3,4^ and covering over 40 protein-coding genes. However, smaller deletions using other combinations of LCRs have been identified. In a study of 200 patients, 8% had LCR22-AB deletions, 2% had LCR22-AC deletions, and 2% had a deletion from downstream of LCR22-A to the typical start of LCR22-D.^4^ Rarer deletion sizes have also been observed.^5^

The clinical presentation of 22q11 patients is remarkably variable. Nevertheless, the major clinical characteristics of the syndrome are velopharyngeal abnormalities, congenital heart anomalies, a characteristic facial appearance and learning disabilities.^6^ In addition, patients with 22q11 deletions are at significant risk for psychiatric disorders, including up to 30% developing schizophrenia.^7^ The syndrome is the second most common cause of intellectual disability, accounting for ~2.4% of patients with a developmental delay.^8^ However, none of these features appear to be fully penetrant, and each exhibits variable expression. One potential mechanism for this phenotypic variability could reside in the variation present in the remaining allele. The hemizygous deletion could unmask recessive variants on the other allele, giving rise to a pathogenic phenotype.^9,10^ For example, hemizygous mutations in SNAP29 can confer cerebral dysgenesis, neuropathy, ichthyosis and keratoderma, Kousseff, or a potentially autosomal recessive form of Opitz G/BBB syndrome.^11^ In addition, hemizygous mutations in GP1BB were initially proposed and later demonstrated to cause Bernard–Soulier syndrome.^9,12^

Sorting out which variants do and do not influence phenotype in our genomes is the next great challenge for human geneticists.^13^ The main approach to map pathogenic variation is to link or associate variants with disease phenotypes. Mapping heterozygous variation in population studies of normal individuals is usually not sufficient to know whether or not a variant can be disease causing, as only a minority of disorders are dominant. One approach to map benign variation is via the analysis of variation present in large-scale genotyping studies of homozygous variants, including nonsense variants, and null alleles in populations of ‘normal’ individuals.^14,15^ Mapping the variation in the remaining alleles of genomic deletion disorders might well be another rich resource to annotate the human genome. Determining a repository of variants identified and the phenotypes accrued is a first step towards the annotation of variation in 22q11. Here we catalog the variation present in the remaining allele in 127 patients with 22q11 deletions.

## Materials and methods

### Sample collection

Subjects were recruited through genetics centers in Europe and the United States. Informed consent was obtained from all subjects, and the study was approved by the appropriate Institutional Review Boards.

### Capture design and sequencing

Custom designed Nimblegen 12 plex arrays (NimbleGen, Inc., Madison, WI, USA) were used to capture the 22q11.2 region for 72 patients. A separate cohort of 55 patients was captured using a custom Agilent Sureselect design (Agilent Technologies, Santa Clara, CA, USA), also targeting the 22q11.2 region. Sequencing was performed on an Illumina HiSeq 2000 and FASTQ files (Illumina, Inc., San Diego, CA, USA) generated with the standard Illumina pipeline. All reads have been deposited into the ENA.^16^

### Variant and deletion identification

Reads were aligned and variants called with an in-house pipeline using Samtools v0.1.18^17^, BWA v0.6.2^18^ for alignment (option -q 15) to the reference human genome (GRCh37 as from 1000 genome project^19^), Picard v1.78^20^ to mark duplicates and GATK v2.4.9^21–23^ for realignment, base score recalibration, and variant calling (UnifiedGenotyper, options -dcov 200 --output_mode EMIT_ALL_SITES --genotype_likelihoods_model BOTH). Variant annotation was performed with Annovar v2013Feb11.^24^ In addition, positions of conserved transcription factor-binding sites and ENCODE^25,26^ ChIP-seq regions were annotated using UCSC Table^27–29^ downloads. Variant positions from segmental duplications^30,31^ (UCSC Table download) were filtered out. The Phred-scaled likelihoods field was used to filter out likely reference position calls (reference likelihood 0, homozygous variant likelihood >70) and indeterminable calls (all likelihoods⩽70). After identifying deletion sizes (see below), heterozygous variant calls were removed from the hemizygous region by only keeping positions with a homozygous variant likelihood equal to zero and reference likelihood >70.

**Figure 1:**
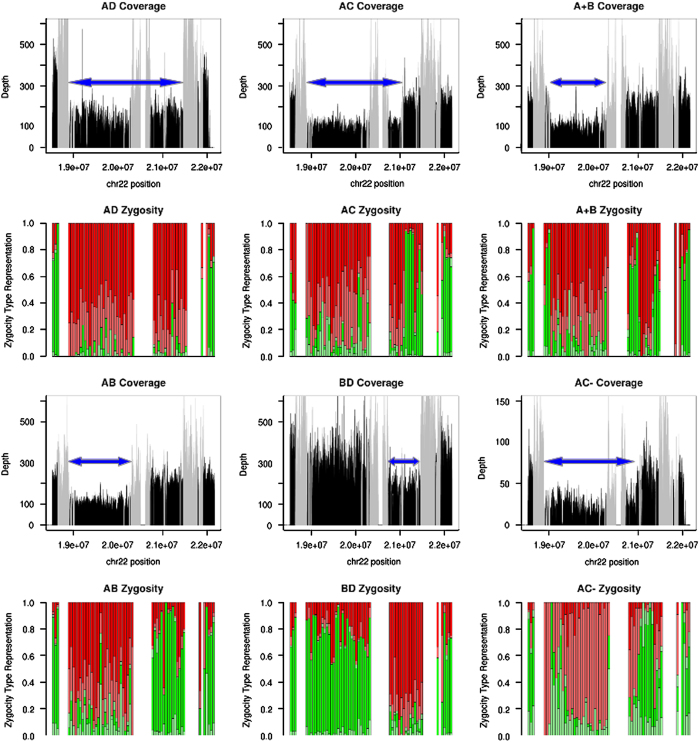
Plots of coverage and zygosity for example LCR22-AD, LCR22-AB, LCR22-AC and LCR22-BD deletions, as well as the LCR22-A^+^B and LCR22-AC^−^ patients. Blue arrows illustrate deletion sizes. For coverage, the maximum depth displayed is 600, except the low-coverage LCR22-AC^−^ sample is set to 150, and segmental duplications are masked in gray. For zygosity plots, within 50 kb windows is indicated the percent of variants using the vcf file's Phred-scaled likelihood (PL) field as follows: bright red=confident homozygous variant (homozygous variant likelihood 0, others >70), light red=less confident homozygous variant (homozygous variant likelihood 0, heterozygous likelihood ⩽70), bright green=confident heterozygous variant (heterozygous likelihood 0, others >70), light green=less confident heterozygous variant (heterozygous likelihood 0, one of the others ⩽70).

The deletion sizes, and hence the location of hemizygous variants, were determined using sequencing depths for each patient across all basepairs in chr22:18520000–22170500. These were extracted from bam files using SNIFER (E. Souche, personal communication). Basepairs in segmental duplications were flagged and all basepairs converted to coverage in 100 bp bins with custom scripts, followed by creating plots in R. Besides coverage, additional support was provided by plots of the percent of variant call types (see Figure 1 legend) in 50 kb windows.

To determine whether variant calls differed between capture platforms, sequences composed of LCR22-AD deletion variant positions were made using homozygous reference calls, homozygous variant calls, or otherwise just called N. These were used as input for MEGA5.10^32^ and unweighted pair group method with arithmetic mean (pairwise-deletion) was performed to create a phylogenic tree.

### Variants per gene

The expected number of variants per gene was determined by first identifying non-sequenceable bases, defined as positions with only 0 or 1 reads in >75% of samples, as well as positions overlapping segmental duplications. This was done to eliminate regions that were difficult to capture or sequence. Hemizygous variants were then counted in the capture data in exonic, coding, or intronic basepairs, as defined by hg19 Refseq, as well as the number of variants between genes (i.e., intergenic regions). Variants for 1000 Genome^19^ data were also obtained by downloading the chromosome 22 phase 1 vcf file from the 1000 Genomes ftp site^33^ and similarly analyzed for the number of variants from sequenceable bases per exonic, coding, intronic or intergenic category. Plots of sequenceable length versus the number of variants were created in R and a best linear fit determined. Outliers were identified as points with Studentized residuals <−2 or >2.

The average conservation score for each gene (exonic or coding basepairs) was also determined by first downloading UCSC Comparative Genomics Conservation scores for chromosome 22 based on multiple alignments of 99 vertebrate genomes to the human genome.^34,35^ Similar to the above analysis, segmental duplications and regions that were difficult to capture or sequence were filtered out. The average conservation score was then calculated per exonic or coding basepair for hg19 Refseq genes in the region.

Genes were annotated for known features based on literature searches and web-based OMIM queries.^36^ In addition, genes were annotated for homozygous knockout phenotypes in mouse models according to the Mouse Genome Informatics WebSite.^37,38^

## Results

### Deletion size

Samples were obtained for 127 patients, targeted captures performed (55 using Agilent Sureselect capture and 72 with custom Nimblegen arrays), reads aligned and variants called. The deleted region per patient was determined based on plots of coverage and heterozygosity (Figure 1), identifying in total 8 LCR22-AB, 2 LCR22-AC, 111 LCR22-AD and 4 LCR22-BD deletions. Besides identifying common LCR-mediated breakpoints, two deletions were identified with atypical breakpoints. One deletion resembles an LCR22-AB deletion, but starts further downstream of LCR22-A (Figure 1). This is a deletion start downstream of LCR22-A similar to that reported in Shaikh *et al.*,^4^ but with the distal rearrangement in LCR22-B. Another patient was also found to have an LCR22-AC resembling deletion, but the deletion ends before LCR22-C (Figure 1). These two deletions were labeled LCR22-A^+^B and LCR22-AC^−^, respectively. Coverage plots did not indicate any large deletion events on the remaining allele.

### Hemizygous variant overview

Across all patients, a total of 18,153 variant positions were initially identified in the hemizygous regions. However, upon closer scrutiny of these positions, 11.6% of Nimblegen and 24.3% of Agilent capture variants per patient were on average called heterozygous. As only a single allele is present, heterozygous variants in this region represent either mosaicisms in the original sample, or are technical artifacts. At the cost of removing potential true positives, a conservative approach was taken and heterozygous calls in the hemizygous region were removed from further analysis, leaving 11,913 variant positions (Supplementary File S1). Removal of heterozygous calls increased the percent of previously annotated variant positions from 57.8% (10,493 of 18,153 variant positions) to 84% (9,990 of 11,913 variant positions). More variants were identified per sample in DNA captured with the Nimblegen capture design as compared with the Agilent design (Supplementary Figure S1). However, when clustering hemizygous variants there was no segregation by capture technology (Supplementary Figure S2).

**Figure 2:**
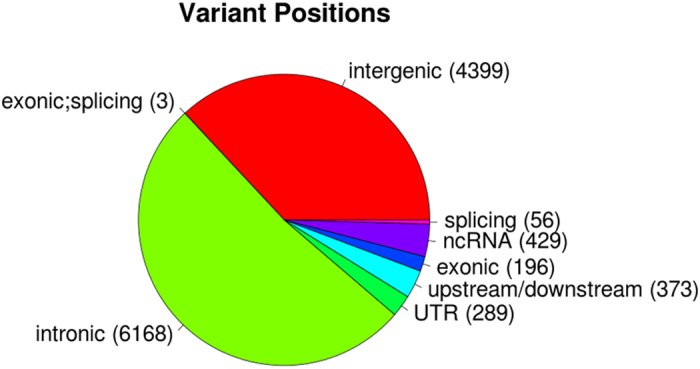
Refseq based variant positions. In parenthesis is indicated the number of variant positions with corresponding annotation.

As larger deletions likely harbor an increased number of variants the average number of variants per patient are segregated by deletion type: 1201 LCR22-AB, 1317 LCR22-AC, 1538 LCR22-AD, 572 LCR22-BD, 587 LCR22-AC^−^ and 835 LCR22-A^+^B variants (Supplementary Figure S1). As defined by Refseq, these are primarily located in introns (52%), due to the gene rich nature of the non-repetitive basepairs in the region, and intergenic regions (37%; Figure 2). Of those, 30 variants are within a conserved transcription factor-binding site and 22 variants are in ENCODE transcription factor ChIP-seq regions. A total of 1.7% of the variants (199) are located in the coding regions. A total of 44% (88) of coding variant positions are synonymous, 48% (95) non-synonymous, two insertions resulted in a frameshift and three variants resulted in a stop-gain.

Filtering for rare variants, defined as a frequency ⩽5% in 1000 genome data and in the sequenced 22q11 patient data set, identified 2 rare stop gains, 1 rare frameshift insertion and 63 rare non-synonymous variants. The rare frameshift insertion was found in a single patient, the rare stop gains across 5 patients, and the rare non-synonymous variants across 55 patients. In total, 25 genes across 57 patients carry a rare protein-altering variant.

### Toleration of gene variation and nullisomy

**Figure 3:**
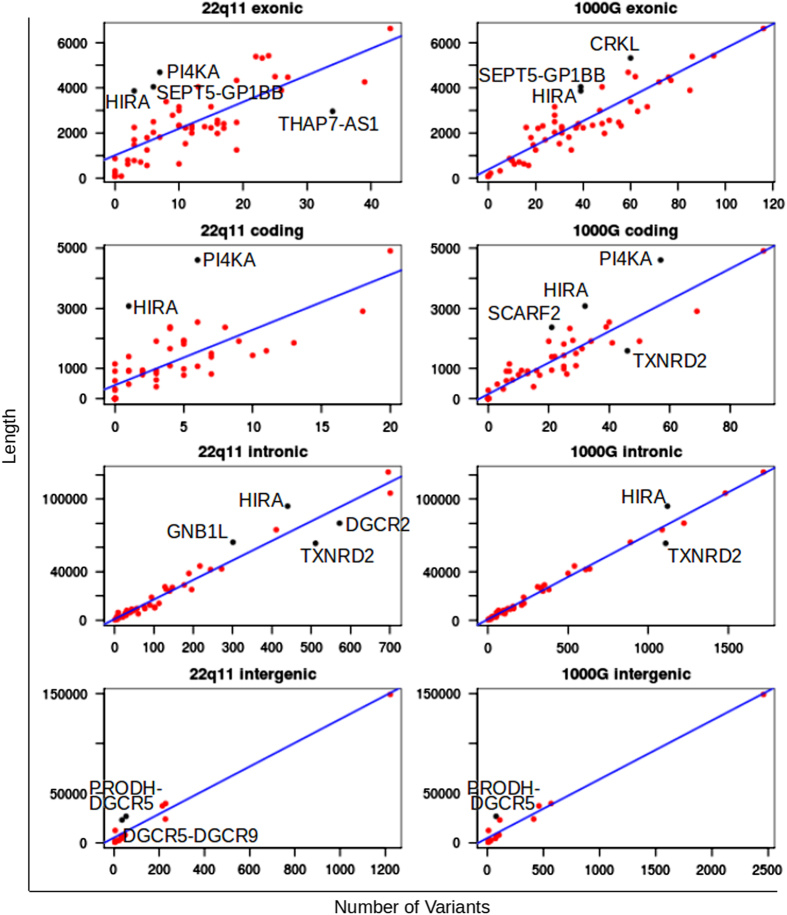
For the 22q11 cohort of this study and 1000 genome data is plotted the number of variants found versus sequenceable length for Refseq defined genes (i.e., exonic), coding region per gene, intronic regions per gene, and intergenic regions. Blue lines are a best linear fit and black points indicate outliers.

To determine if some genes in the region were more or less tolerant to variation, we identified genes with high and low conservation. In particular, within these 22q11 data and in 1000 genome data HIRA and PI4KA had fewer variants than expected (Figure 3), indicating these genes are more conserved and potentially less tolerant to variation. Fittingly, in the 22q11 patients HIRA had no variants affecting the open reading frame. Additional support for these genes having low tolerance to variation is that they also have high average cross-species conservation scores (Table 1).

Mutations that alter protein structure potentially affect function. Overall, protein-altering mutations were identified in 28 genes (Table 2). Seven of these genes are known for involvement in human autosomal recessive syndromes and 19 genes have been demonstrated to be embryonic lethal or result in defective phenotypes in mouse models (Table 2).

## Discussion

Many genomic disorders can result in a broad phenotypic spectrum, despite having similar sized deletions or duplications. For deletions, it has been speculated that variation in the remaining allele might explain part of this phenotypic variability.^9,10^ Although sporadic analysis of the remaining allele has been instigated, thus far, no comprehensive analysis of a single genomic disorder has been performed to test this hypothesis. Hemizygous variant alleles present only in patients with a specific phenotype are likely to be the cause of such phenotypic differences. In addition, hemizygous alleles with variable or no phenotypic expression are likely to be benign. Considering the remarkable phenotypic variation observed for 22q11DS, we set out to explore the extent of variation in those patients, identifying a total of 11,913 hemizygous variant positions. Rare protein-altering variants are found in 57 patients and in 25 of 40 genes (Table 2).

On the basis of knockout mouse models, embryonic or neonatal lethality has been shown for eight genes in the 22q11 region (Table 2). Interestingly, here we identified non-synonymous and stop-gain mutations in six out of these eight genes. This suggests either these genes are not lethal in humans, or more likely these mutations result in partially functional proteins. Rare non-synonymous variants are found in CLDN5, CDC45, DGCR8, GNB1L and PI4KA. Four of these genes have disease associations, including all having been associated with schizophrenia.^39–42^ A stop-gain was identified in a single patient in TBX1. TBX1 is known to be responsible for several of the major 22q11 deletion syndrome phenotypes, including abnormal facies (conotruncal anomaly face), cardiac defects, thymic hypoplasia, velopharyngeal insufficiency with cleft palate and parathyroid dysfunction with hypocalcaemia.^43^ This stop-gain removes the last 19 amino acids of exon 9A of a minor isoform of the gene.^44^ Though TBX1 as a whole is considered essential for life, this variant proves that at least the last 19 amino acids of this isoform are not.

Patients with microdeletion syndromes are at an increased risk to manifest autosomal recessive disorders.^9^ Currently seven protein-coding genes within the 22q11.2 deletion region have been annotated to cause autosomal recessive conditions in humans (Table 2). Interestingly, half of these genes have protein-altering mutations in our cohort: PI4KA, PRODH, SCARF2 and SNAP29. Two non-synonymous variants (rs151146863 and chr22:21224655 C>T) and a frameshift insertion (chr22:21224770 T>TAG) were each found in SNAP29 in single previously reported individuals^11^ and in no additional patients in our cohort. On the basis of their atypical phenotypes (Supplementary Table S1), this demonstrates that mutations on the non-deleted chromosome can lead to unmasking of autosomal recessive conditions such as cerebral dysgenesis, neuropathy, ichthyosis and keratoderma, Kousseff, and a potentially autosomal recessive form of Opitz G/BBB syndrome.^11^ Notably only two genes with embryonic lethality in mice show low mutation load in this cohort: HIRA and PI4KA (Tables 1 and 2, Figure 3). Fittingly, in this cohort we did not identify any variants affecting the HIRA open reading frame. For PI4KA we did identify two rare non-synonymous positions (rs61752248 in two patients and previously unannotated chr22:21081649 G>C in a single patient with schizophrenia). The function of this gene is not well recognized, but PI4KA has previously been associated with schizophrenia, perisylvian polymicrogyria, cerebellar hypoplasia and arthrogryposis.^39,47^ In addition to its role in psychiatric disorders and in brain development, these results indicate PI4KA is an essential gene for human life.

Thus far 18 genes in the 22q11 region have not been associated with specific phenotypes in man or mice. Nevertheless, 11 of these genes have been identified with rare variants. Further evaluation of the patients carrying these variants may identify genotype–phenotype relationships, leading to future knowledge of gene functions.

In conclusion, we have created an extensive catalog of 22q11 hemizygous variation. These variants begin to provide insight into phenotypic contributions for the genes in the region, as well as tolerability of 22q11 gene variation and nullisomy. This catalog will serve as a blueprint for future experiments to correlate 22q11DS variation with phenotype and provides as an example for the challenges linking diverse phenotypes with large numbers of variants as we move from targeted to genome-wide sequencing.

## Figures and Tables

**Table 1:** Top 10 highest average gene conservation scores

*Gene (Exonic Nts)*	*Score*	*Gene (coding Nts)*	*Score*
MIR3618	7.44385	DGCR8	4.99448
MIR1306	6.76083	UFD1L	4.86823
PI4KA	4.66779	LZTR1	4.77470
ZDHHC8	4.56044	PI4KA	4.75533
HIRA	3.87669	RANBP1	4.70058
CDC45	3.73804	CRKL	4.63475
CLTCL1	3.70709	ZDHHC8	4.56044
RANBP1	3.63031	HIRA	4.48574
DGCR8	2.95486	SLC25A1	4.27646
SLC25A1	2.95161	DGCR2	4.13267

**Table 2:** Genes, cohort variants (rare in parenthesis) and annotation

*Gene*	*Percent sequenced (coding, %)*	*Stop gain*	*Frameshift insertion*	*Non-synonymous*	*OMIM & literature annotated features*	*Homozygous KO mice*
AIFM3	100	—	—	9 (8)	—	—
ARVCF	100	—	—	11 (8)	22Q^a^	abnormal gait and cataract
C22orf29	100	—	—	3 (3)	—	—
C22orf39	100	—	—	—	—	—
CDC45	100	—	—	1 (1)	—	embryonic lethal
CLDN5	100	1 (0)	—	1 (1)	—	blood-brain barrier loosening, premature neonatal lethality
CLTCL1	99	—	1 (0)	14 (9)	—	—
COMT	100	—	—	1 (0)	22Q^a^, S^a^	increased dopamine levels in male frontal cortex. Behavioral changes
CRKL	100	—	—	—	22Q^a^, #115470. CAT EYE SYNDROME^a^, Conotruncal Heart Defects (CTDs)^b^^,^^45^	—
DGCR14	100	—	—	4 (3)	—	—
DGCR2	100	—	—	2 (1)	22Q^a^, S^a^	—
DGCR6L	0	—	—	—	—	—
DGCR8	100	—	—	2 (2)	22Q^a^, S^a^	embryonic lethal
GNB1L	100	—	—	5 (3)	22Q^a^	embryonic lethal
GP1BB	45	—	—	—	22Q^a^, #231200. BERNARD-SOULIER SYNDROME (caused by homozygous or compound heterozygous mutation)^a^	giant platelets, severe bleeding
GSC2	52	—	—	—	—	normal
HIRA	100	—	—	—	22Q^a^	disrupted embryonic development, embryonic lethal
KLHL22	100	—	—	—	—	—
LOC388849	0	—	—	—	—	—
LZTR1	100	—	—	1 (1)	#615670. SCHWANNOMATOSIS 2; SWNTS2 (autosomal dominant inheritance and incomplete penetrance)^a^, Noonan syndrome^b^^,^^46^	—
MED15	100	—	—	1 (1)	—	—
MRPL40	100	—	—	2 (0)	—	—
P2RX6	100	—	—	1 (0)	—	increased thermal response latency, resistant to metrazol-induced seizures
PI4KA	72	—	—	2 (2)	Perisylvian polymicrogyria, cerebellar hypoplasia and arthrogryposis (compound heterozygous)^b^^,^^47^	embryonic lethal
PRODH	22	1 (1)	—	2 (1)	22Q^a^, S^a^, #239500. HYPERPROLINEMIA, TYPE I (autosomal recessive)^a^	reduced male body weight, hyperprolinemia, increased startle reflex, and regionally altered brain levels of multiple amino acids
RANBP1	98	—	—	—	22Q^a^	growth retardation, decreased body weight, male infertility
RIMBP3	0	—	—	—	—	male infertility
RTN4R	98	-	—	—	S^a^	impaired behavior and coordination, improved spinal cord regeneration
SCARF2	90	—	—	8 (2)	#600920. VAN DEN ENDE-GUPTA SYNDROME (autosomal recessive)^a^	—
SEPT5	96	—	—	—	#231200. BERNARD-SOULIER SYNDROME (homozygous or compound heterozygous mutation)^a^	synaptic transmission defects for one allele; platelet secretion and behavioral defects reported for a different allele
SERPIND1	100	—	—	4 (4)	—	normal
SLC25A1	86	—	—	—	22Q^a^, #615182. COMBINED D-2- AND L-2-HYDROXYGLUTARIC ACIDURIA (homozygous or compound heterozygous mutations)^a^	—
SLC7A4	100	—	—	4 (2)	—	—
SNAP29	100	—	1 (1)	2 (2)	#609528. CEREBRAL DYSGENESIS, NEUROPATHY, ICHTHYOSIS, AND PALMOPLANTAR KERATODERMA SYNDROME (homozygous mutation)^a^	—
TANGO2	100	—	—	1 (1)	—	—
TBX1	78	1 (1)	—	2 (0)	22Q^a^, #217095. CONOTRUNCAL HEART MALFORMATIONS; #187500. TETRALOGY OF FALLOT^a^	neonatal lethality, abnormal blood vessel and ear development, and abnormal cranial base morphology
THAP7	100	—	—	2 (1)	—	—
TMEM191B	0	—	—	—	—	—
TRMT2A	100	—	—	3 (2)	—	—
TSSK2	100	—	—	3 (2)	—	male infertility
TXNRD2	100	—	—	—	22Q^a^	embryonic lethal
UFD1L	100	—	—	1 (1)	—	normal
ZDHHC8	32	—	—	—	S^a^	behavioral changes
ZNF74	100	—	—	3 (2)	22Q^a^	—

a =OMIM annotations.

b =Literature annotations.

For OMIM annotations, 22Q=#608363 CHROMOSOME 22q11.2 DUPLICATION SYNDROME, #188400 DIGEORGE SYNDROME, and/or #192430 VELOCARDIOFACIAL SYNDROM. S=#181500. SCHIZOPHRENIA and/or #600850. SCHIZOPHRENIA 4. Mouse homozygous knockout descriptions have been shortened. For full descriptions see the Mouse Genome Informatics WebSite.^37,38^
