# Supplementary material for: A catalog of hemizygous variation in 127 22q11 deletion patients
Source: Hum Genome Var. 2016 Jan 14;3:15065–. doi: 10.1038/hgv.2015.65 (PMC4892188; doi:10.1038/hgv.2015.65)
Supplement: Supplementary Information [file hgv201565-s1.doc]

## Supplemental Files:

**Figure S1:** Number of hemizygous variants per patient, separated by deletion type. Red bars indicate samples using Agilent capture technology and purple bars indicate samples using Nimblegen capture technology. (TIFF)

**Figure S2:** Clustering of hemizygous variant positions for all LCR22-AD deletion samples. KUL samples are Nimblegen captures and Emory samples are Agilent captures. (pdf)

**Table S1:** The number of rare protein altering variants and rare patient phenotypes per gene. Not all patients had extensive phenotyping, so it should be noted that missing phenotypes for a gene/patient does not indicate a phenotype is not present. (pdf)

**File S1:** Hemizygous variant positions as a tab delimited text file.
