# Supplementary figures and images for: A catalog of hemizygous variation in 127 22q11 deletion patients
Source: Hum Genome Var. 2016 Jan 14;3:15065–. doi: 10.1038/hgv.2015.65 (PMC4892188; doi:10.1038/hgv.2015.65)

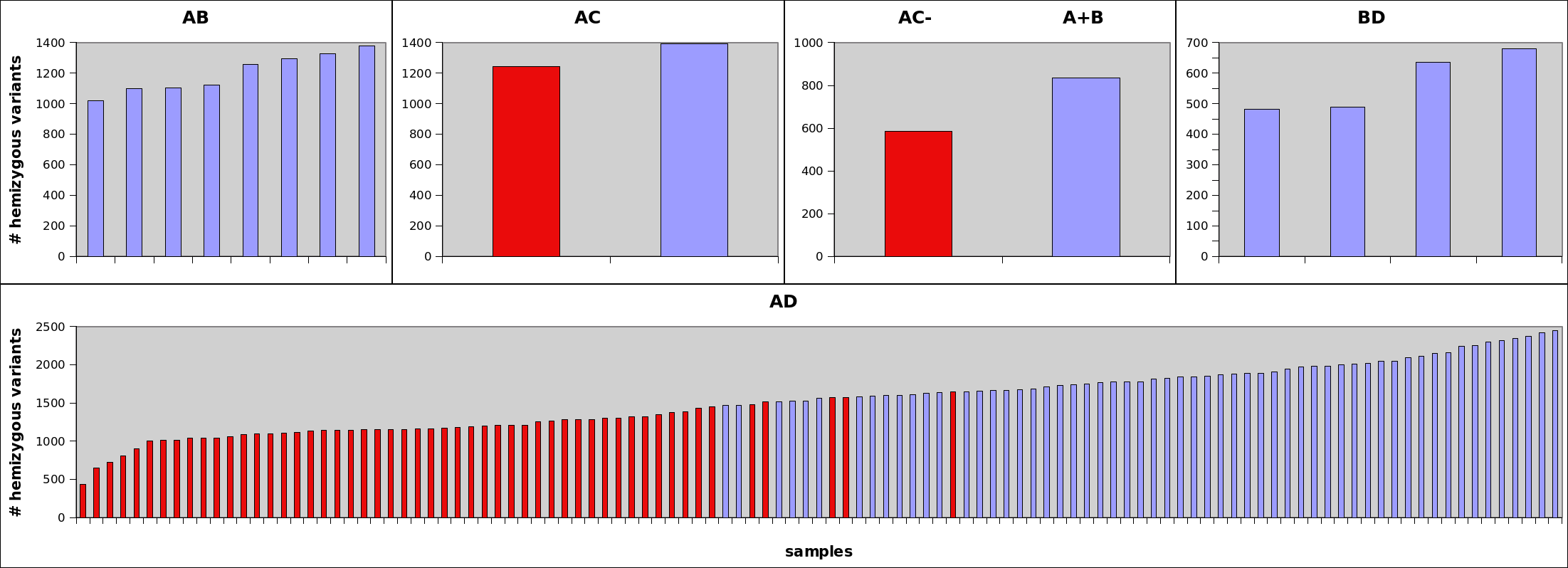

Supplement: Supplementary Figure S1 [file hgv201565-s3.tiff]

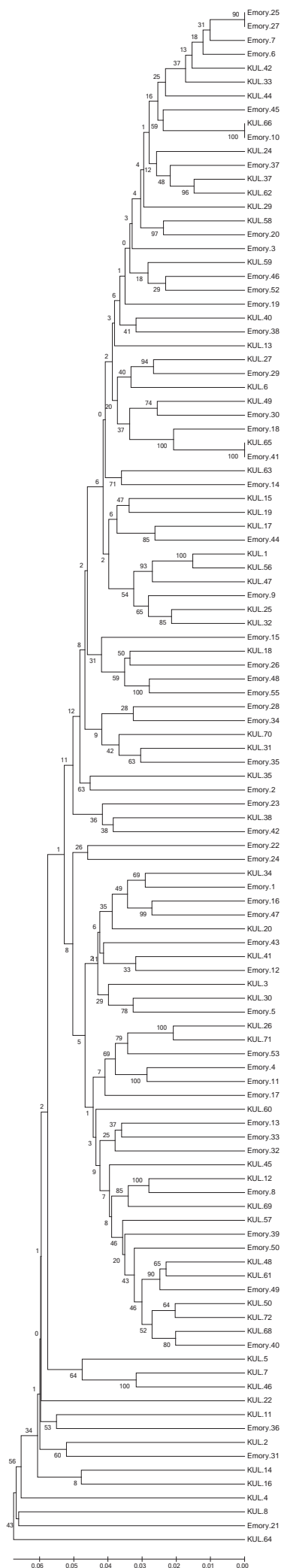

Supplement: Supplementary Figure S2 [file hgv201565-s4.pdf]
