# Supplementary material for: A catalog of hemizygous variation in 127 22q11 deletion patients
Source: Hum Genome Var. 2016 Jan 14;3:15065–. doi: 10.1038/hgv.2015.65 (PMC4892188; doi:10.1038/hgv.2015.65)
Supplement: Supplementary Table S1 [file hgv201565-s5.pdf]

Gene	Rare protein altering variants	Atypical Phenotypes per Patient (P)
AIFM3	8 non-synonymous	P1 Evans Syndrome, bilateral postaxial polydactyly of hands and feet, bilateral inguinal hernias; P2 Polydactyly (bilateral postaxial hands, unilateral preaxial foot); P6 anorectal malformation; P49 laryngeal web
ARVCF	8 non-synonymous	P7 Camptodactyly IV; P50 unilateral cryptorchism; P25 myringotomy tubes
C22orf29	3 non-synonymous	P19 Bilateral club feet; P51 micropenis; P52 Ehlers Danlos Syndrome; P54 L5S1 myelomenigocele, Hydrocephalus; P43 growth hormone deficiency, cerebral palsy
CDC45	1 non-synonymous	P41 Cleft lip and palate
CLTCL1	9 non-synonymous	P1 Evans Syndrome, bilateral postaxial polydactyly of hands and feet, bilateral inguinal hernias; P13 Polymicrogyria, Laryngomalacia, keratoderma; P36 Hashimoto's thyroiditis, congenital scoliosis
DGCR14	3 non-synonymous	P30 Hashimoto's thyroiditis; P53 subglottic stenosis, cerebral palsy, congenital dislocation of the hip.
DGCR2	1 non-synonymous	P36 Hashimoto's thyroiditis, congenital scoliosis
GNB1L	3 non-synonymous	P17 Bilateral iris coloboma; P41 cleft lip and palate
PRODH	1 non-synonymous, 1 stop gain	P19 Bilateral club feet; P38 intestinal malrotation
SCARF2	2 non-synonymous	P38 Intestinal malrotation
SERPIND1	4 non-synonymous	P1 Evans Syndrome, bilateral postaxial polydactyly of hands and feet, bilateral inguinal hernias; P17 bilateral iris coloboma
SLC7A4	2 non-synonymous	P21 Polymicrogyria, NMR: bifrontal subependymal heterotopia
SNAP29	2 non-synonymous, 1 frameshift insertion	P22 Myelomeningocele, hydrocephalus, subglottic stenosis; P13 Polymicrogyria, Laryngomalacia, keratoderma; P41 cleft lip and palate
TBX1	1 stop gain	P23 Congenital anal atresia
TRMT2A	2 non-synonymous	P17 Bilateral iris coloboma; P29 Pierre-Robin anomalad, idiopathic thrombocytopenia, intestinal malrotation, growth hormone deficiency; P41 cleft lip and palate
TSSK2	2 non-synonymous	P1 Evans Syndrome, bilateral postaxial polydactyly of hands and feet, bilateral inguinal hernias
UFD1L	1 non-synonymous	P6 Anorectal malformation
ZNF74	2 non-synonymous	P24 Double aortic arch; P35 Idiopathic thrombocytopenia, bilateral club feet
